# Effect of Drying Temperature and Storage Time on the Fatty Acid Composition and Lipid Indices in Wild Edible King Bolete Mushroom (*Boletus edulis*)

**DOI:** 10.3390/molecules31162855

**Published:** 2026-08-15

**Authors:** Marek Šnirc, Ivona Jančo, Silvia Jakabová, Jozef Golian, Mirosław Mleczek, Július Árvay

**Affiliations:** 1 Institute of Food Sciences, Faculty of Biotechnology and Food Sciences, Slovak University of Agriculture, Tr. A. Hlinku 2, 949 76 Nitra, Slovakia; marek.snirc@uniag.sk (M.Š.); silvia.jakabova@uniag.sk (S.J.); jozef.golian@uniag.sk (J.G.); 2 Agrobiotech—Research Centre, Slovak University of Agriculture, Tr. A. Hlinku 2, 949 76 Nitra, Slovakia; ivona.janco@uniag.sk; 3 Department of Chemistry, Poznań University of Life Sciences, Wojska Polskiego 75, 60-625 Poznań, Poland; miroslaw.mleczek@up.poznan.pl

**Keywords:** *Boletus edulis*, fatty acid composition, drying temperature, storage duration, lipid indices

## Abstract

Drying and storage are widely used to preserve edible mushrooms, yet information on how different drying temperatures and prolonged storage affect the relative fatty acid profile of wild *Boletus edulis* remains limited. This study aimed to determine the effects of drying temperature (20–60 °C), lyophilization, and storage duration (up to 6 months) on the fatty acid methyl ester (FAME) profile and fatty acid profile-derived indices of *B. edulis*. FAMEs were analyzed by GC-FID and expressed as relative percentages of total identified FAMEs. Linoleic acid predominated (58.7–70.1%), followed by oleic and palmitic acids. Samples dried at 40–60 °C showed higher relative PUFA proportions, approaching those of lyophilized samples. During storage, SFA increased from 12.9% to 16.2%, whereas PUFA decreased from 65.9% to 59.7%. Exploratory multivariate analysis suggested visual grouping of samples according to drying temperature, mainly related to differences in MUFA, SFA, and PUFA proportions. The highest PUFA/SFA ratio was 5.54. Overall, drying conditions and storage duration were associated with shifts in the relative fatty acid profile of *B. edulis*. Because total lipid content and direct oxidation markers were not measured, these results describe compositional differences and do not demonstrate absolute lipid retention, degradation, or oxidation.

## 1. Introduction

Edible mushrooms are valued for their sensory properties and for the presence of several nutritionally relevant compounds. Although mushrooms generally contain low amounts of total lipids, their fatty acid profiles are often dominated by unsaturated fatty acids, particularly linoleic and oleic acids [1,2,3]. Therefore, fatty acid analysis in mushrooms is useful mainly for characterizing the composition and post-harvest stability of the lipid fraction rather than for estimating a major contribution to dietary lipid intake [4,5].

The genus *Boletus*, and particularly *Boletus edulis*, includes some of the most appreciated edible wild mushrooms because of their organoleptic quality and nutritional characteristics [6,7]. In *B. edulis*, linoleic acid is generally reported as the predominant fatty acid, followed by oleic and palmitic acids, while unsaturated fatty acids usually represent the major fraction of the total identified fatty acids [8,9]. More specifically, linoleic acid (C18:2n6c), oleic acid (C18:1n9c), and palmitic acid (C16:0) are the principal contributors to the relative PUFA, MUFA, and SFA proportions, respectively. Because these acids account for most of the identified fatty acid methyl ester (FAME) profile, their relative proportions provide useful compositional indicators of drying- and storage-related shifts and contribute substantially to the values of the profile-derived indices evaluated in this study. This fatty acid pattern is characteristic of a PUFA-dominated relative fatty acid profile in *B. edulis*; however, this interpretation should be considered in the context of the low total lipid content of mushrooms.

Mushroom foraging is a common activity in Slovakia, and wild edible mushrooms are frequently preserved for later consumption [10]. Species such as *B. edulis*, *Cantharellus cibarius*, and members of the genera *Leccinum* and *Boletus* are among the most frequently collected and highly valued mushrooms because of their culinary and nutritional properties [11]. Traditional preservation practices, including drying and pickling, remain part of local dietary habits and are also relevant from the perspective of food quality, safety, and nutrient retention during storage [12]. From a practical perspective, information on how commonly used drying temperatures and subsequent storage affect the relative fatty acid profile is relevant to mushroom producers, processors, and consumers when evaluating preservation conditions and the compositional quality of dried mushrooms.

The lipid profile of mushrooms is influenced by intrinsic factors, including species and maturity stage, as well as by extrinsic factors such as post-harvest handling, drying conditions, and storage regime [13,14]. Drying is one of the most widely used preservation methods for wild mushrooms because it reduces water activity, extends shelf life, and limits microbial spoilage. At the same time, drying may induce temperature-dependent changes in the relative fatty acid composition of mushrooms [13,15]. Subsequent storage can further modify the fatty acid profile, with the extent and direction of these changes depending on storage duration, residual moisture, temperature, oxygen availability, and the characteristics of the mushroom matrix [5,16].

Despite the recognized nutritional and analytical relevance of mushroom lipids, the available literature does not provide a fully consistent picture of how drying and storage affect fatty acid composition. Some studies have reported lower proportions of polyunsaturated fatty acids (PUFAs), relative increases in saturated fatty acids (SFAs), or shifts in the monounsaturated fatty acid/polyunsaturated fatty acid balance after thermal processing or storage [1,5,8,15]. These changes are often discussed in relation to possible oxidation-related processes. However, direct confirmation of lipid oxidation requires specific markers, such as peroxide value, thiobarbituric acid reactive substances, or conjugated dienes; changes in relative fatty acid proportions alone are not sufficient to confirm oxidation.

In contrast, other studies have reported relatively preserved fatty acid distributions or higher relative proportions of unsaturated fatty acids under selected drying conditions [13,17,18,19]. Such outcomes may be related to faster moisture removal, shorter exposure to conditions that promote lipid changes, and earlier inactivation of oxidative enzymes. These apparently divergent findings may reflect differences in mushroom species, drying intensity and duration, storage conditions, residual moisture, and analytical expression of the results. In particular, interpretation differs substantially depending on whether the data are expressed as relative fatty acid proportions or as absolute lipid contents.

This issue is especially relevant for *B. edulis*, which is commonly preserved by drying and stored for later consumption. Oancea et al. [13] previously examined changes in the fatty acid and aroma profiles of *B. edulis* after hot-air and centrifugal-vacuum dehydration, while other studies characterized the fatty acid composition of *B. edulis*, related Boletaceae, and edible mushrooms more broadly [14,20,21,22]. However, information remains limited on changes in the relative fatty acid methyl ester profile and fatty acid profile-derived indices when wild *B. edulis* collected in Slovakia is subjected to drying at 20–60 °C, with lyophilization as a reference treatment, followed by storage for up to six months under controlled post-harvest conditions. Accordingly, the contribution of the present study lies in the comparative evaluation of this defined combination of processing and storage conditions rather than in the first characterization of the fatty acid composition of the species. A better understanding of these effects is needed not only for analytical characterization of processed mushroom products but also for a more restrained nutritional interpretation of lipid indices calculated from relative fatty acid data.

In this context, the present study aimed to evaluate the effects of drying temperature and storage duration on the fatty acid methyl ester profile and related lipid indices of wild edible *B. edulis*. We hypothesized that both drying temperature and storage time would significantly influence the relative fatty acid composition and the derived indices. Rather than assuming complete stability of the lipid profile, we expected that prolonged storage and different drying conditions would be associated with shifts in the relative proportions of fatty acid classes. Because lipid changes during drying and storage may result from complex and potentially non-linear processes, we further assumed that these changes would not necessarily follow a strictly linear pattern.

## 2. Results

### 2.1. Fatty Acid Methyl Ester Profile of Boletus Edulis

A total of 14 fatty acid methyl esters (FAMEs) were identified in the analyzed Boletus edulis samples. The relative fatty acid profile was dominated by unsaturated fatty acids, with linoleic acid (C18:2n6c) as the predominant fatty acid. Its relative proportion ranged from 58.7 to 70.1% of the total identified fatty acids. Oleic acid (C18:1n9c) was the main monounsaturated fatty acid and ranged from 14.4 to 24.9%, while palmitic acid (C16:0) was the dominant saturated fatty acid, with values between 11.1 and 12.2%.

Stearic acid was detected at lower relative proportions, ranging from 1.09 to 2.10%. Myristic acid was present at 0.17–0.21%. Minor fatty acids, including pentadecanoic acid, heptadecanoic acid, arachidic acid, and gamma-linolenic acid, occurred only at low or trace levels. Gamma-linolenic acid was detected in the range from not detected to 0.08%. Elaidic acid, the trans isomer of oleic acid, was also present only in small amounts, ranging from 0.02 to 0.17%.

When individual fatty acids were grouped into the main fatty acid classes, polyunsaturated fatty acids (PUFA) represented the predominant fraction, accounting for approximately 65% of the relative fatty acid profile. Monounsaturated fatty acids (MUFA) represented approximately 20%, while saturated fatty acids (SFA) accounted for approximately 15%. These values indicate that the lipid fraction of *B. edulis* was characterized by a high relative proportion of PUFA, mainly due to the dominance of linoleic acid. Table 1 presents the ranges of individual fatty acids observed in the present study within the broader ranges reported for mushrooms. In particular, the high relative PUFA proportion was mainly attributable to linoleic acid (58.7–70.1%), whereas oleic acid (14.4–24.9%) accounted for most of the MUFA proportion.

### 2.2. Effect of Drying Temperature on SFAs, MUFAs, and PUFAs

**Figure 1:**
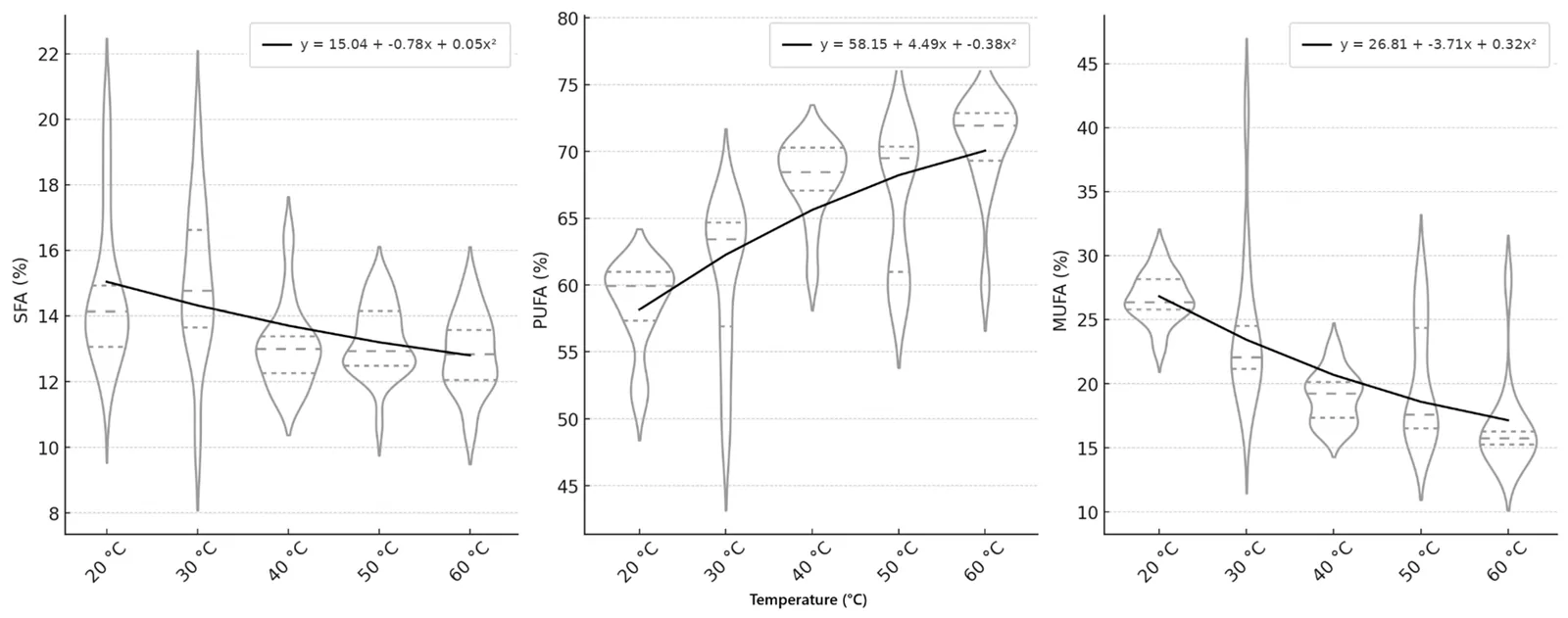
Visualization of SFA, MUFA, and PUFA proportions across drying temperatures in *B. edulis.* The fitted second-order polynomial curves and equations are presented solely as descriptive visual aids and were not used for statistical inference or prediction.

Drying temperature significantly affected the relative distribution of SFAs, MUFAs, and PUFAs in *B. edulis*. The Kruskal–Wallis test indicated statistically significant differences among drying temperature treatments for all three fatty acid classes (*p* < 0.0001). Subsequent Dunn–Holm comparisons identified significant pairwise differences among the hot-air drying temperatures, as indicated by the superscript letters in Table 2. The descriptive statistics are presented in Table 2, and the distribution of SFAs, MUFAs, and PUFAs across drying temperatures is visualized in Figure 1. The fitted polynomial curves were included only to provide a descriptive visualization of possible non-linear patterns in the observed distributions and were not used for inferential interpretation. The lyophilized sample is presented as a reference control and was not considered a hot-air-drying temperature treatment.

At lower and moderate drying temperatures, particularly 20 and 30 °C, samples showed a higher relative proportion of MUFA than samples dried at higher temperatures. MUFA values reached approximately 24.0–26.9% under these drying conditions. In contrast, increasing drying temperature was associated with a higher relative proportion of PUFA and a lower relative proportion of MUFA. The highest PUFA proportion was observed at 60 °C, where PUFA represented up to 70.3% of the total identified fatty acids. The relative PUFA proportion in samples dried at 60 °C approached the values observed in lyophilized samples.

The SFA fraction showed only minor variation across the drying temperature range. SFA values fluctuated between approximately 12.8 and 13.8%, indicating that the applied drying temperatures affected mainly the relative balance between MUFA and PUFA, while SFA remained comparatively stable.

### 2.3. Effect of Storage Duration on SFAs, MUFAs, and PUFAs

**Figure 2:**
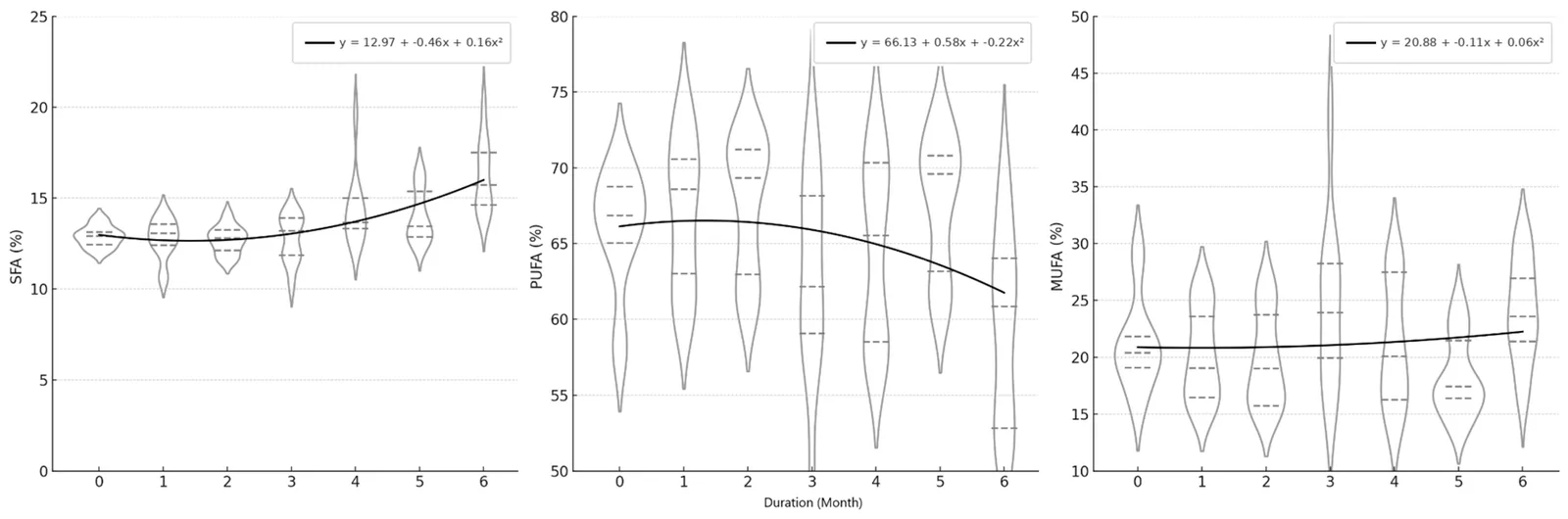
Visualization of SFA, MUFA, and PUFA proportions across storage duration in *B. edulis*. The fitted second-order polynomial curves and equations are presented solely as descriptive visual aids and were not used for statistical inference or prediction.

Storage duration also significantly affected the relative distribution of SFA, MUFA, and PUFA in *B. edulis*. The Kruskal–Wallis test confirmed statistically significant differences among storage times for all three fatty acid classes (*p* < 0.0001). Subsequent Dunn–Holm comparisons identified significant pairwise differences among storage months, as indicated by the superscript letters in Table 3. The descriptive statistics are summarized in Table 3, and the corresponding distributions and temporal patterns of SFA, MUFA, and PUFA are shown in Figure 2.

During the six-month storage period, the relative proportion of SFA increased from 12.9 ± 0.57% at month 0 to 16.2 ± 1.98% after six months. This increase indicates a storage-related shift in the relative fatty acid profile toward a higher proportion of saturated fatty acids.

MUFA showed a variable pattern during storage and did not follow a clear monotonic trend. Although differences among storage times were observed, MUFA changes fluctuated during the storage period and were less directionally consistent than the changes observed for SFA and PUFA.

PUFA remained the dominant fatty acid class throughout the whole storage period and showed a variable pattern over time. However, its relative proportion was lower after six months than at the beginning of storage, decreasing from 65.9 ± 3.93% at month 0 to 59.7 ± 6.16% after six months. Thus, storage was associated with a lower relative proportion of PUFA and a corresponding higher relative proportion of SFA at the end of the storage period.

Although statistically significant differences were detected among storage times, the polynomial regression models showed low explanatory power. Therefore, the fitted curves are presented solely as descriptive visual aids for possible non-linear patterns in the observed distributions and should not be interpreted as inferential or predictive models of storage-related fatty acid changes.

### 2.4. Exploratory Principal Component Analysis of Fatty Acid Composition as Affected by Drying Temperature and Storage Duration

Principal component analysis (PCA) was used as an exploratory multivariate approach to assess relationships among fatty acid variables and to visualize sample grouping according to drying temperature and storage duration. The first two principal components explained 59.3% of the total dataset inertia. Dimension 1 accounted for 41.0% of the variance, while Dimension 2 explained an additional 18.2%. The distribution of samples in the PCA space suggested visual grouping according to drying temperature; however, this pattern was interpreted descriptively and not as inferential evidence of group separation.

The PCA biplots showed that sample distribution was mainly associated with differences in the relative proportions of SFA, MUFA, and PUFA. Positive coordinates on Dimension 1 were associated with higher relative proportions of SFA, including C14:0, C15:0, C16:0, C17:0, C18:0, and C20:0, and selected MUFA, including C14:1, C18:1n9c, and C20:1c. In contrast, samples with lower Dimension 1 scores were associated with higher relative proportions of PUFA, particularly C18:2n6c.

**Figure 3:**
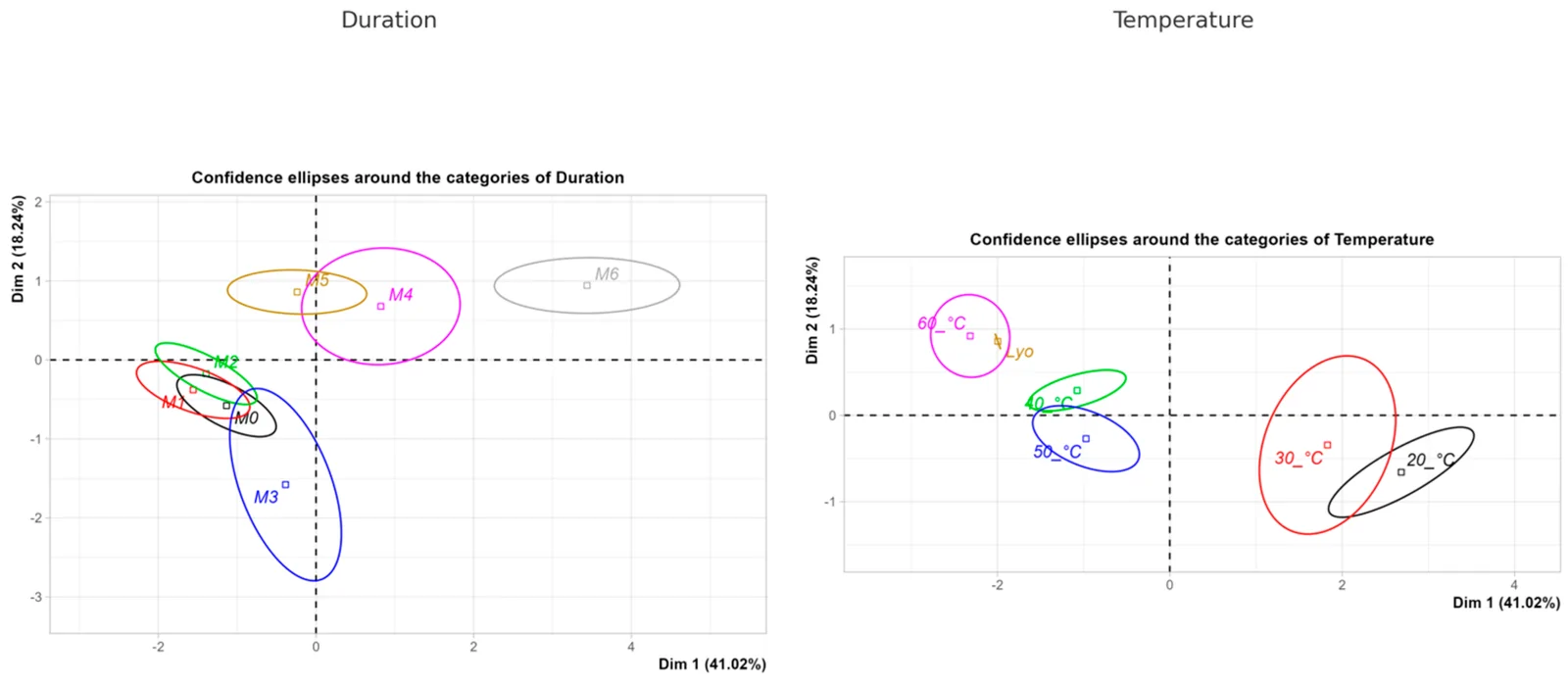
Exploratory PCA score plots of fatty acid profiles in *B. edulis* samples with confidence ellipses illustrating within-group dispersion and overlap according to storage duration (**left**) and drying temperature (**right**).

Dimension 2 (Figure 3) further described variation in sample distribution associated with fatty acid class composition. Positive values on Dimension 2 were associated with selected unsaturated fatty acids, particularly C18:2n6c and C18:3n6c, together with the monounsaturated fatty acids C16:1 and C17:1c, whereas negative values were associated with higher relative proportions of SFA and selected MUFA.

**Figure 4:**
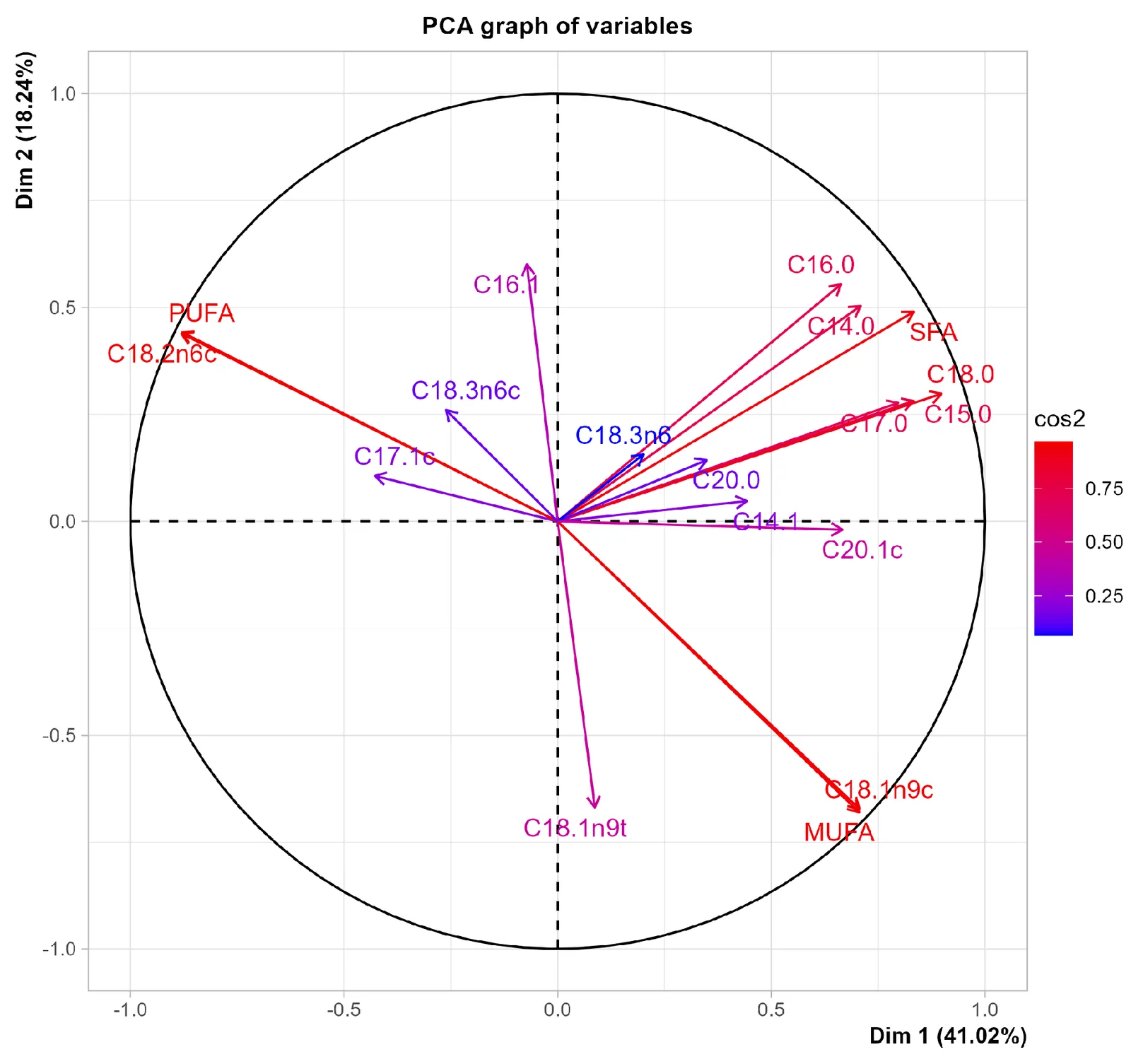
Principal component analysis loading plot of fatty acid variables in *B. edulis*, showing their associations with the first two principal components.

Samples dried at higher temperatures were positioned closer to variables associated with higher PUFA proportions, particularly linoleic acid. In contrast, samples dried at lower temperatures were more closely associated with higher relative proportions of SFA and MUFA. A visual tendency related to storage duration was also observed, with later storage stages positioned closer to variables representing higher SFA proportions. Samples from the early storage stages showed greater overlap in the PCA space (Figure 4).

### 2.5. Fatty Acid Profile-Derived Indices in B. edulis as Affected by Drying Temperature and Storage Duration

Fatty acid profile-derived indices were calculated to compare the relative fatty acid distribution in *B. edulis* under different drying and storage conditions. The evaluated indices included PUFA/SFA, index of atherogenicity (IA), index of thrombogenicity (IT), hypocholesterolemic/hypercholesterolemic ratio (HH), unsaturation index (UI), and total trans fatty acids (TFA). Significant pairwise differences among storage months and drying temperatures are indicated by superscript letters in Table 4 and Table 5, respectively.

During storage, the PUFA/SFA ratio decreased from 5.13 ± 0.37 at month 0 to 3.77 ± 0.80 after six months (Table 4). This decrease reflected the storage-related reduction in the relative proportion of PUFA and the corresponding increase in SFA. In parallel, IA increased from 0.13 ± 0.01 to 0.17 ± 0.02, and IT increased from 0.29 ± 0.02 to 0.37 ± 0.05. The HH ratio decreased from 7.76 ± 0.51 to 6.30 ± 0.65 during the same period.

The unsaturation index decreased from 153 ± 3.90 at month 0 to 143 ± 7.97 after six months. TFA values remained low throughout storage. The highest mean TFA value was observed at month 3 (0.32 ± 0.75), but this parameter showed high relative variability and remained at trace levels.

Drying temperature was also associated with changes in the calculated lipid indices (Table 5). The highest PUFA/SFA ratio was recorded at 60 °C (5.54 ± 0.62), whereas the lowest value was observed at 20 °C (4.10 ± 0.68). These values correspond to the higher relative PUFA proportions detected in samples dried at elevated temperatures.

IA and IT remained relatively stable in numerical terms across the applied drying temperatures, although selected pairwise differences were statistically significant (Table 5). HH reached the highest values at 40 °C (7.63 ± 0.63) and 50 °C (7.46 ± 0.61). The unsaturation index increased with increasing drying temperature, from 144 ± 4.99 at 20 °C to 157 ± 3.94 at 60 °C. TFA values remained low under all drying conditions, with the lowest mean values recorded at elevated temperatures (0.02 ± 0.03), but no significant pairwise differences were detected among the hot-air drying temperatures.

## 3. Discussion

The present study confirmed that the fatty acid profile of *B. edulis* is dominated by unsaturated fatty acids, with linoleic acid as the major fatty acid, followed by oleic and palmitic acids. This pattern is consistent with previous reports describing *B. edulis* and other edible mushrooms as matrices in which linoleic and oleic acids usually predominate within the identified lipid fraction [7,8,9,13,20]. The relative proportions observed in the present study fall within the wide ranges reported for edible mushrooms, confirming that the lipid profile of mushrooms is highly species-dependent and may also vary according to geographical origin, environmental conditions, maturity stage, morphological part of the fruiting body, and differences in lipid extraction, derivatization, and chromatographic quantification [7,8,9,14,21,22,23]. More specifically, Polish *B. edulis* samples contained 32.78–50.62% linoleic acid, 19.56–44.75% oleic acid, and 8.39–28.84% palmitic acid [7], compared with 58.7–70.1%, 14.4–24.9%, and 11.1–12.2%, respectively, in the present study. Ribeiro et al. [9] additionally reported oleic acid as the predominant fatty acid in Portuguese *B. edulis*. Within Boletaceae, linoleic acid predominated in *B. appendiculatus* and *X. chrysenteron*, whereas oleic acid was prominent in *B. edulis* and *B. impolitus* [8]. From a compositional perspective, the predominance of linoleic acid largely determines the PUFA fraction, while its role as a precursor of C8 volatile compounds, including 1-octen-3-ol, also makes it relevant to the characteristic mushroom aroma [13]. Oleic acid, as the principal MUFA, accounts for most of the MUFA fraction; therefore, the relative balance between linoleic and oleic acids strongly influences the overall degree of unsaturation and the profile-derived indices evaluated in this study.

The dominance of PUFA in the present samples characterizes the relative fatty acid distribution within the lipid fraction of *B. edulis*. However, this interpretation requires caution. Mushrooms generally contain low total lipid amounts; therefore, relative fatty acid proportions should not be interpreted as evidence of a substantial contribution to total dietary lipid intake [4,5]. In this respect, the present results are most appropriately understood as a compositional characterization of the lipid fraction and as an assessment of how this fraction responds to drying and storage, rather than as a direct nutritional effect.

Drying temperature was one of the major factors associated with differences in the relative fatty acid profile. Samples dried at higher temperatures, particularly 40–60 °C, showed higher relative PUFA proportions and lower MUFA proportions than samples dried at lower temperatures. This agrees with the findings of Oancea et al. [13], who reported changes in oleic and linoleic acid proportions in *B. edulis* after dehydration treatments. At first, the higher relative PUFA proportion at elevated drying temperatures may seem counterintuitive, because PUFA are generally more susceptible to oxidation than SFA. However, the present data are expressed as relative proportions of identified fatty acids, not as absolute fatty acid contents. Consequently, the observed increase in PUFA proportion does not necessarily indicate an absolute increase in PUFA, but rather a redistribution within the fatty acid profile. These relative data therefore do not allow conclusions regarding the absolute retention or loss of individual fatty acids, total lipid degradation, or lipid oxidation.

A possible explanation for the pattern observed at higher drying temperatures is that faster moisture removal may have shortened the period during which mushroom tissues remained at intermediate moisture levels that may favor enzymatic or oxidative changes. Higher drying temperatures may also have contributed to earlier inactivation of endogenous enzymes involved in lipid transformation. Similar mechanisms have been discussed in relation to dehydration dynamics and enzyme inactivation in food matrices [17,18,19]. Nevertheless, these explanations remain mechanistic hypotheses. Because peroxide value, TBARS, conjugated dienes, p-anisidine value, or other direct lipid oxidation markers were not determined, oxidation cannot be confirmed from the present dataset and is considered only as a possible explanation for the observed relative compositional changes.

The storage experiment showed a storage-related shift in the relative fatty acid profile by the end of the six-month period, characterized mainly by a higher relative proportion of SFAs and a lower relative proportion of PUFAs compared with month 0. This trend is consistent with previous studies reporting storage- or processing-related changes in mushroom lipid profiles, including lower relative PUFA proportions and higher relative SFA proportions under selected conditions [1,5,15,24,25,26]. The lower PUFA proportion observed after six months is compatible with the general susceptibility of unsaturated fatty acids to changes during storage but does not demonstrate oxidative degradation. However, as in the drying experiment, the absence of direct oxidation markers prevents a definitive attribution of these changes to lipid oxidation. Other factors, including residual moisture, oxygen availability, matrix heterogeneity, packaging conditions, and storage temperature, may have contributed to the observed pattern.

The statistical evaluation further indicates that storage duration should not be interpreted as a single deterministic predictor of fatty acid changes. Although significant differences among storage times were detected, the low explanatory power of the polynomial regression models suggests that the temporal pattern was not strictly linear and that storage time alone explained only a limited proportion of the total variability. This supports the initial assumption that lipid profile changes during drying and storage may result from complex and potentially non-linear processes. Therefore, the statistically significant storage effect should be interpreted as evidence of group-wise differences among storage periods, not as evidence of a robust predictive temporal model.

The PCA supported the univariate findings by showing that the separation of samples was mainly associated with differences in SFA, MUFA, and PUFA proportions. Samples dried at higher temperatures were positioned closer to variables associated with higher PUFA proportions, particularly linoleic acid, whereas lower-temperature drying was more closely associated with higher MUFA and SFA proportions. The PCA also indicated that samples from later storage stages were more strongly associated with increased SFA proportions, while samples from early storage stages were less clearly separated. This exploratory pattern was consistent with the univariate finding that drying temperature and storage duration were associated with differences in the relative fatty acid dataset.

The calculated fatty acid profile-derived indices provided an integrated view of these compositional changes. The decrease in the PUFA/SFA ratio during storage reflected the relative decline in PUFA and the relative increase in SFA. Similarly, the modest increases in IA and IT and the decrease in HH were driven by shifts in the relative proportions of fatty acids included in the numerator and denominator of these equations. Such indices are commonly used to compare fatty acid profiles among food matrices [4,16,26,27]. In *B. edulis*, these values provide comparative information on the relative fatty acid distribution and are consistent with the previously reported compositional characteristics of this species [6,7,27]. However, these indices should not be overinterpreted as direct indicators of a health effect, because they were calculated from relative fatty acid percentages and not from absolute lipid intake.

Drying temperature also influenced the calculated indices. The highest PUFA/SFA ratio was observed at 60 °C, corresponding to the highest relative PUFA proportion recorded under the applied drying conditions. The relatively stable IA and IT values across drying temperatures indicate that the applied drying treatments did not markedly shift the balance toward fatty acids included in the atherogenicity or thrombogenicity equations. The higher UI values at elevated drying temperatures reflected the greater degree of unsaturation within the relative fatty acid profile. However, as with PUFA/SFA, UI should be interpreted as a comparative descriptor of the lipid fraction rather than as evidence of a quantitatively higher intake of unsaturated fatty acids.

The very low TFA values observed in the present study are also relevant from a compositional perspective. Elaidic acid and total trans fatty acids occurred only at trace levels, and no consistent drying- or storage-related pattern was evident. Therefore, the TFA data should be interpreted cautiously and primarily as confirmation that trans fatty acids represented only a minor component of the identified fatty acid profile under the applied processing and storage conditions.

From a practical perspective, the present results indicate that drying temperature and storage duration can influence the relative fatty acid profile of dried *B. edulis*. Drying at 40–60 °C was associated with higher relative PUFA proportions and corresponding changes in the calculated lipid indices compared with lower drying temperatures. However, these findings should not be interpreted as a universal recommendation for all drying conditions. Drying efficiency, residual moisture, sensory quality, storage stability, and consumer acceptability must also be considered when selecting technological conditions for mushroom preservation.

Several limitations should be considered. First, the samples originated from one locality and one harvesting period, which limits the generalization of the results to all *B. edulis* populations. Second, the data were expressed as relative fatty acid proportions, and absolute lipid content was not quantified. Third, no direct lipid oxidation markers were measured, which limits the mechanistic interpretation of PUFA and SFA changes. Fourth, the storage experiment simulated domestic storage in closed glass jars at room temperature and in the dark. Residual moisture content, water activity after drying and during storage, as well as headspace volume, and oxygen concentration were not quantified, which limits assessment of their possible contribution to storage-related changes in the relative fatty acid profile. Therefore, the findings may not apply to vacuum packaging, modified atmosphere packaging, refrigeration, or other storage systems. Future studies should combine relative fatty acid profiling with absolute lipid quantification, residual moisture or water activity measurements, oxygen exposure assessment, and direct oxidation markers. Such an approach would allow a more precise interpretation of whether the observed changes represent true lipid degradation, redistribution within the lipid fraction, or matrix-dependent analytical variation.

Overall, the findings support the working hypothesis that both drying temperature and storage duration significantly influence the relative fatty acid composition and fatty acid profile-derived indices of *B. edulis*. The changes were not strictly linear and should be interpreted as the result of interacting technological and matrix-related factors. The study therefore contributes to a more nuanced understanding of changes in the relative fatty acid profile of dried wild mushrooms and emphasizes that lipid indices calculated from mushroom fatty acid profiles should be interpreted as comparative descriptors rather than direct nutritional or health claims.

## 4. Materials and Methods

### 4.1. Mushroom Material and Sampling Area

Fresh fruiting bodies of the king bolete mushroom, *Boletus edulis* Bull. Fr., were collected in October 2019 in the cadastral territory of the village of Liptovská Lúžna, Slovakia. The sampling site is located in the Lúžňanská Valley in the northern part of the Low Tatras, covering an area of approximately 5466 ha at elevations ranging from 700 to 850 m a.s.l. The region is characterized by a cold climate and average annual precipitation of approximately 1000–1200 mm, creating favorable conditions for mushroom growth.

A total of 3.5 kg of fresh fruiting bodies was collected for the experiment. Only visually healthy fruiting bodies without visible mechanical damage or signs of decay were included. After collection, the samples were transported to the laboratory and processed prior to drying. Comparable sampling approaches based on visually healthy wild fruiting bodies and prompt pre-analytical processing have been used in compositional studies of *B. edulis* and related Boletaceae mushrooms [7,14].

### 4.2. Drying, Lyophilization, and Storage Experiment

The collected mushroom samples, consisting exclusively of fruiting bodies, were sliced into uniformly sized pieces to ensure consistent drying. The prepared slices were dried in a hot-air drying oven Memmert UF 110m (Memmert GmbH & Co. KG, Schwabach, Germany) at 20, 30, 40, 50, and 60 °C until constant mass was reached after approximately 24 h. The temperature range of 20–60 °C was selected to represent low-to-moderate hot-air drying conditions relevant to domestic mushroom preservation. Temperatures of 70–80 °C were not included because preliminary observations during method development indicated pronounced undesirable changes in mushroom color and aroma. Residual moisture content and water activity were not determined after drying or during storage; constant mass was therefore used as the operational drying endpoint. The selected temperature range was chosen to simulate the most common hot-air drying conditions used in domestic mushroom preservation. Higher temperatures, such as 70–80 °C, were not included because preliminary observations showed that such drying conditions markedly impaired the sensory quality of the mushrooms, particularly color, aroma, and overall acceptability.

In parallel, a control sample was processed by lyophilization. For this purpose, mushroom slices were cut into approximately 1 × 1 cm cubes and shock-frozen at −80 °C. Lyophilization was carried out using a LyoQuest freeze dryer (Telstar SLU, Barcelona, Spain) at −80 °C for 48 h. Lyophilized samples were used as a reference control for comparison with hot-air-dried samples, whereas the storage-duration experiment was evaluated for the hot-air-dried samples.

After drying, the samples were stored for six months at room temperature (22 ± 2 °C) in closed 700 mL glass jars sealed with metal lids and kept in a dark room to simulate typical domestic storage conditions. The dried mushroom material was not weighed before storage; instead, each jar was filled to its nominal capacity of 700 mL. The jars were filled and sealed under ambient laboratory conditions without active control of the oxygen atmosphere. Although filling the jars to capacity minimized the headspace above the samples, atmospheric air remained in the interstitial spaces, and neither the headspace volume nor the oxygen concentration was quantified. The use of hot-air drying and lyophilization is consistent with previous dehydration studies on *B. edulis* and other mushrooms [13,15], while storage at room temperature is comparable to long-term storage experiments conducted with dried *B. edulis* at 20 °C [27]. Samples were collected and analyzed at monthly intervals over the six-month storage period. Separate jars were assigned to the individual storage intervals and remained unopened until their scheduled analysis; therefore, no jar was repeatedly opened or resampled across storage times. At each monthly sampling point, 20 independent storage units (separate, previously unopened jars) were evaluated. For each drying temperature condition, four independent samples were analyzed, and all analytical determinations were carried out in triplicate. Fatty acid composition was expressed as the relative percentage (%) of individual fatty acids within the total identified fatty acids, and the results are presented as mean values ± standard deviation.

### 4.3. Extraction and Determination of Fatty Acid Methyl Esters

The homogenized dried mushroom samples were extracted using a modified protocol based on the methods of Bligh and Dyer [28] and Folch et al. [29]. For esterification of sample lipids to fatty acid methyl esters (FAMEs), approximately 0.2 g of sample was placed in a 15 mL plastic vial and mixed with 7.6 mL of a chloroform:methanol:ultra-pure water mixture (2:4:1.6, *v*/*v*/*v*). Chloroform (ChromaSolv, ≥99.9%; Honeywell, Seelze, Germany), methanol (ChromaSolv, ≥99.9%; Sigma-Aldrich GmbH, Steinheim, Germany), and ultra-pure water were used for the extraction. The mixture was shaken for 3 h using a Unimax 2010 laboratory shaker (Heidolph GmbH, Schwabach, Germany).

The mixture was subsequently centrifuged at 2236× *g* for 2 min using a Hettich Universal 320 centrifuge (Hettich GmbH, Tuttlingen, Germany) and immediately filtered by filtration under reduced pressure using a vacuum manifold. Thereafter, 1 mL of chloroform, 1 mL of potassium chloride solution (2 mol/L), and 0.9 mL of ultra-pure water were added to each sample, and the samples were centrifuged at 800× *g* for 5 min. The lower organic phase was collected and dried using an SPE vacuum manifold (Agilent Technologies Inc., Santa Clara, CA, USA) in the presence of anhydrous sodium sulfate.

For methylation, 1.45 mL of the dried extract was transferred to an Eppendorf tube and mixed with 50 µL of methanolic potassium hydroxide (2 mol/L) and 500 µL of hexane (SupraSolv, GC purity; Merck KGaA, Darmstadt, Germany). The mixture was vortexed, and 1 mL of the upper clear layer was carefully transferred into a gas chromatography vial prior to chromatographic analysis.

Fatty acids were determined by gas chromatography with flame ionization detection (GC-FID) using an Agilent 7890A gas chromatograph (Agilent Technologies, USA). One microlitre of each sample was injected using a CombiPAL autosampler (CTC Analytics AG, Zwingen, Switzerland). Separation of FAMEs was achieved on an HP-88 capillary column (100 m × 0.25 mm × 0.20 μm; Agilent Technologies, USA). Data acquisition and processing were performed using Agilent OpenLab ChemStation LTS 01.11software. Instrument calibration was carried out using a certified 37-component FAME Mix standard (TraceCERT, Supelco Inc., Bellefonte, PA, USA). For additional qualitative confirmation, a 7890B GC system coupled with a 5977A mass detector (Agilent Technologies, USA) was used.

### 4.4. Fatty Acid Profile-Derived Indices

Selected fatty acid profile-derived indices were calculated from the relative fatty acid profile of *B. edulis* to characterize the distribution of fatty acid classes under different drying and storage conditions. Given the low total lipid content of mushrooms, these indices were interpreted primarily as comparative descriptors of compositional differences within the fatty acid profile. The evaluated indices included the polyunsaturated to saturated fatty acid ratio (PUFA/SFA), index of atherogenicity (IA), index of thrombogenicity (IT), hypocholesterolemic/hypercholesterolemic ratio (HH), unsaturation index (UI), and total trans fatty acid (TFA) content. All indices were calculated based on quantified FAMEs obtained through gas chromatographic analysis of total lipid extracts.

The calculations were performed according to Chen and Liu [4] and Gałgowska and Pietrzak-Fiećko [7], with necessary adjustments for the fatty acid composition of the mushroom matrix.

The PUFA/SFA ratio was used to describe the balance between polyunsaturated and saturated fatty acids within the relative fatty acid profile:

(1) $$PUFA/SFA=\frac{\sum PUFA}{\sum SFA}$$

The index of atherogenicity was calculated as follows:

(2) $$IA=\frac{C12:0+\left(4\times C14:0\right)+C16:0}{\sum UFA}$$

where C12:0, C14:0, and C16:0 represent the percentages of lauric, myristic, and palmitic acids, respectively, and ΣUFA represents the total content of unsaturated fatty acids. IA was used as a comparative index reflecting the relative contribution of selected saturated fatty acids and unsaturated fatty acids within the lipid fraction.

The index of thrombogenicity was calculated according to the equation:

(3) $$IT=\frac{C14:0+C16:0+C18:0}{\left(0.5\times \sum MUFA\right)+\left(0.5\times \sum n-6PUFA\right)+\left(3\times \sum n-3PUFA\right)+(\frac{n-3}{n-6})}$$

where C14:0, C16:0, and C18:0 represent the percentages of myristic, palmitic, and stearic acids, respectively; ΣMUFA represents the total content of monounsaturated fatty acids; and Σn-6 PUFA and Σn-3 PUFA represent the total contents of n-6 and n-3 polyunsaturated fatty acids, respectively.

The hypocholesterolemic/hypercholesterolemic ratio was calculated as follows:

(4) $$HH=\frac{cis-C18:1+\sum PUFA}{C14:0+C16:0+C18:0}$$

where cis-C18:1 represents the percentage of cis-oleic acid, ΣPUFA represents the total content of polyunsaturated fatty acids, and C14:0, C16:0, and C18:0 represent the contents of myristic, palmitic, and stearic acids, respectively.

The unsaturation index was calculated by summing the products of the percentage of each unsaturated fatty acid and its number of double bonds:

(5) UI=(1×%monoenoic)+(2×%dienoic)+(3×%trienoic)+…

This index reflects the degree of unsaturation within the relative fatty acid profile. Total trans fatty acids were calculated as the sum of all identified trans isomers and expressed as their relative occurrence within the total identified fatty acids.

### 4.5. Statistical Analysis

The normality of data distribution was first evaluated using the Shapiro–Wilk test. Because the data did not satisfy the assumptions of normality, non-parametric statistical methods were used in all subsequent analyses. Descriptive statistics were reported as median, mean, and standard deviation. The Kruskal–Wallis test was applied to assess the overall effects of drying temperature and storage duration on the studied variables.

Following a significant Kruskal–Wallis result for a given variable, pairwise comparisons were performed using Dunn’s post hoc test with Holm adjustment for multiple comparisons. Significant pairwise differences are indicated by different superscript letters in Table 2, Table 3, Table 4 and Table 5. The lyophilized reference was presented descriptively and excluded from pairwise comparisons among hot-air drying temperatures. Second-order polynomial curves were fitted solely as descriptive visual aids for possible non-linear patterns in the observed data. Because the models showed low explanatory power, they were not used for hypothesis testing, prediction, or identification of drying temperature or storage duration as predictive factors. Inferential interpretation was based primarily on the Kruskal–Wallis test, followed by Dunn’s test where applicable.

To complement the univariate assessment, principal component analysis (PCA) was performed as an exploratory multivariate technique to evaluate relationships among fatty acid variables and to visualize sample grouping according to drying temperature and storage duration. The active variables comprised the relative proportions of all individual fatty acids listed in Table 1 together with the aggregate SFA, MUFA, and PUFA fractions. Before PCA, all active variables were mean-centered and scaled to unit variance. Each row of the PCA matrix represented one sample-level fatty acid profile, whereas drying temperature and storage duration were used only as supplementary categorical variables for grouping and visualization and were not included as active variables in the calculation of the principal components. Confidence ellipses were used only to illustrate within-group dispersion and overlap. PCA has been widely used for the characterization of complex food quality datasets and for identifying variables that contribute most strongly to sample differentiation [30]. The PCA was interpreted exclusively as an exploratory analysis, and no inferential conclusions were drawn from the apparent visual separation of sample groups.

All statistical analyses were performed using RStudio version 2024.09.1 with relevant R packages, including following modules: ggbetweenstats [31], R [32], ggplot2 [33], and dplyr [34].

## 5. Conclusions

The present study showed that drying temperature and storage duration significantly affected the relative fatty acid composition and fatty acid profile-derived indices of wild edible *B. edulis*. The analyzed samples were characterized by a lipid fraction dominated by unsaturated fatty acids, particularly linoleic acid, followed by oleic and palmitic acids. However, because mushrooms are inherently low in total lipid content, these findings should be interpreted primarily as comparative information on the relative fatty acid profile rather than as evidence of a substantial contribution to dietary lipid intake.

Drying at higher temperatures, especially 40–60 °C, was associated with a higher relative proportion of PUFAs and with corresponding differences in selected fatty acid profile-derived indices under the applied experimental conditions. In contrast, prolonged storage was associated with a relative increase in SFAs and a decrease in PUFAs, which was reflected mainly in a lower PUFA/SFA ratio and corresponding changes in lipid indices. These results indicate that both drying conditions and storage duration contribute to compositional shifts within the lipid fraction of dried *B. edulis*.

The calculated lipid indices should be regarded as comparative descriptors of fatty acid distribution within the mushroom lipid fraction rather than as direct indicators of nutritional or health effects. The absence of absolute lipid quantification and direct lipid oxidation markers limits the mechanistic interpretation of the observed changes. Accordingly, the present results cannot demonstrate absolute fatty acid retention or loss, total lipid degradation, or lipid oxidation. Therefore, future studies should focus particularly on linoleic acid (C18:2n6c), oleic acid (C18:1n9c), and palmitic acid (C16:0), as the predominant fatty acids within the PUFA, MUFA, and SFA fractions, respectively, and should combine relative fatty acid profiling with absolute lipid content, residual moisture or water activity, and direct oxidation markers to better explain lipid stability during mushroom drying and storage.

From a practical perspective, these findings provide comparative information for mushroom producers, processors, and consumers when considering drying temperature and storage duration, although technological recommendations must also account for residual moisture, sensory quality, storage atmosphere, and consumer acceptability.

Overall, the findings suggest that drying conditions and storage duration are important factors influencing the stability of the relative fatty acid profile of dried *B. edulis*. The results provide a useful basis for further studies on post-harvest processing of wild edible mushrooms and support a more restrained interpretation of fatty acid profile-derived indices in low-lipid mushroom matrices.

## Figures and Tables

**Table 1:** Contextual comparison of relative fatty acid proportions (%) in mushrooms between the present study and previously published data.

Fatty Acid	% in This Study	% in Other Studies	Reference
Myristic acid (C14:0)	0.17–0.21	0.15–18.3	[9,13,16]
Myristoleic acid (C14:1)	0.03–0.14	0.04–1.79	[9,20]
Pentadecanoic acid (C15:0)	0.20–0.40	0.30–25.3	[9,13,16]
Palmitic acid (C16:0)	11.1–12.2	5.9–39.8	[7,8,9,13,16]
Palmitoleic acid (C16:1)	0.86–0.95	0.21–16.1	[9,13]
Heptadecanoic acid (C17:0)	0.07–0.27	0.05–3.5	[9,16]
cis-10-Heptadecenoic acid (C17:1c)	0.24–0.33	0.04–4.79	[9,20]
Stearic acid (C18:0)	1.09–2.10	0.81–76.8	[7,8,9,13,16]
Elaidic acid (C18:1n9t)	0.02–0.17	0.72–1.32	[20]
Oleic acid (C18:1n9c)	14.4–24.9	17.7–79.0	[7,8,9,13]
Linoleic acid (C18:2n6c)	58.7–70.1	10.8–81.1	[1,7,8,9,13,14,21,22,23]
gamma-Linolenic acid (C18:3n6)	ND–0.08	ND–1.30	[9,20]
Arachidic acid (C20:0)	0.06–0.11	0.06–0.76	[9,13,16]
cis-11-Eicosenoic acid (C20:1c)	0.31–0.54	0.25–1.21	[9,13,20]

**Table 2:** Effect of drying temperature on the percentage profile of SFAs, MUFAs, and PUFAs.

Drying Temperature	SFA	MUFA	PUFA
Lyophilized (Lyo)	13.8 ± 0.02	16.1 ± 0.01	70.1 ± 0.02
20 °C	14.6 ± 2.25 ^a^	26.9 ± 1.92 ^a^	58.5 ± 3.07 ^d^
30 °C	14.9 ± 2.34 ^a^	24.0 ± 5.92 ^b^	61.1 ± 5.56 ^c^
40 °C	13.0 ± 1.26 ^b^	19.0 ± 1.95 ^c^	67.9 ± 2.78 ^b^
50 °C	13.3 ± 1.02 ^b^	20.1 ± 4.45 ^c^	66.6 ± 4.97 ^b^
60 °C	12.8 ± 1.16 ^b^	16.9 ± 3.63 ^d^	70.3 ± 3.6 ^a^
*p* (Kruskal–Wallis)	<0.0001	<0.0001	<0.0001

Note: Lyo, lyophilized reference control. Different superscript lowercase letters within a column indicate significant differences among hot-air drying temperatures according to Dunn’s post hoc test with Holm adjustment (*p* < 0.05); values sharing at least one letter do not differ significantly. The lyophilized reference was excluded from pairwise comparisons among hot-air drying temperatures.

**Table 3:** Effect of storage duration on the percentage profile of SFAs, MUFAs, and PUFAs.

Storage Duration (Months)	SFA	MUFA	PUFA
0 (start)	12.9 ± 0.57 ^d^	21.2 ± 4.04 ^abc^	65.9 ± 3.93 ^ab^
1	12.8 ± 1.09 ^d^	20.2 ± 3.94 ^bc^	66.9 ± 4.81 ^a^
2	12.7 ± 0.75 ^d^	19.8 ± 4.09 ^c^	67.5 ± 4.39 ^a^
3	12.8 ± 1.29 ^cd^	24.6 ± 7.35 ^ab^	62.7 ± 6.91 ^bc^
4	14.4 ± 2.01 ^b^	21.1 ± 5.35 ^abc^	64.6 ± 5.90 ^ab^
5	14.0 ± 1.43 ^bc^	18.3 ± 3.44 ^c^	67.7 ± 4.41 ^a^
6	16.2 ± 1.98 ^a^	24.0 ± 4.46 ^a^	59.7 ± 6.16 ^c^
*p* (Kruskal–Wallis)	<0.0001	<0.0001	<0.0001

Different superscript lowercase letters within the same column indicate significant differences among storage durations according to Dunn’s post hoc test with Holm adjustment (*p* < 0.05); values sharing at least one superscript letter do not differ significantly. The *p*-values correspond to the overall Kruskal–Wallis test.

**Table 4:** Effect of storage duration on lipid indices (PUFA/SFA, IA, IT, HH, UI, TFA) in *B. edulis*.

Storage Duration (Month)	PUFA/SFA	IA	IT	HH	UI	TFA
0 (start)	5.13 ± 0.37 ^ab^	0.13 ± 0.01 ^c^	0.29 ± 0.02 ^c^	7.76 ± 0.51 ^a^	153 ± 3.90 ^ab^	0.07 ± 0.05 ^bc^
1	5.31 ± 0.85 ^a^	0.13 ± 0.01 ^c^	0.28 ± 0.03 ^c^	7.76 ± 0.68 ^a^	154 ± 5.79 ^ab^	ND ^e^
2	5.35 ± 0.57 ^a^	0.13 ± 0.01 ^c^	0.28 ± 0.02 ^c^	7.80 ± 0.49 ^a^	154 ± 4.83 ^a^	ND ^e^
3	4.93 ± 0.62 ^ab^	0.13 ± 0.01 ^c^	0.28 ± 0.03 ^c^	7.73 ± 0.62 ^a^	150 ± 6.71 ^b^	0.32 ± 0.75 ^a^
4	4.59 ± 0.79 ^b^	0.15 ± 0.02 ^b^	0.32 ± 0.05 ^b^	7.11 ± 0.9 ^b^	150 ± 7.04 ^b^	0.02 ± 0.02 ^ac^
5	4.90 ± 0.74 ^ab^	0.15 ± 0.01 ^b^	0.31 ± 0.03 ^b^	7.06 ± 0.62 ^b^	153 ± 5.61 ^ab^	0.01 ± 0.01 ^ad^
6	3.77 ± 0.8 ^c^	0.17 ± 0.02 ^a^	0.37 ± 0.05 ^a^	6.30 ± 0.65 ^c^	143 ± 7.97 ^c^	0.04 ± 0.02 ^b^

Different superscript lowercase letters within the same column indicate significant differences among storage durations according to Dunn’s post hoc test with Holm adjustment (*p* < 0.05); values sharing at least one superscript letter do not differ significantly. ND = not detected.

**Table 5:** Effect of drying temperature on lipid indices (PUFA/SFA, IA, IT, HH, UI, TFA) in *B. edulis*.

Drying Temperature	PUFA/SFA	IA	IT	HH	UI	TFA
Lyo	5.07 ± 0.01	0.15 ± 0.01	0.31 ± 0.01	6.88 ± 0.03	156 ± 0.03	0.11 ± 0.08
20 °C	4.10 ± 0.68 ^c^	0.15 ± 0.02 ^b^	0.33 ± 0.06 ^a^	7.21 ± 0.97 ^a^	144 ± 4.99 ^d^	0.10 ± 0.41 ^a^
30 °C	4.19 ± 0.69 ^c^	0.15 ± 0.02 ^a^	0.34 ± 0.06 ^a^	6.81 ± 0.94 ^b^	146 ± 6.10 ^c^	0.17 ± 0.54 ^a^
40 °C	5.26 ± 0.61 ^ab^	0.14 ± 0.01 ^b^	0.29 ± 0.03 ^b^	7.63 ± 0.63 ^a^	154 ± 3.83 ^b^	0.02 ± 0.02 ^a^
50 °C	5.08 ± 0.69 ^b^	0.14 ± 0.01 ^b^	0.30 ± 0.03 ^b^	7.46 ± 0.61 ^a^	153 ± 5.62 ^b^	0.02 ± 0.02 ^a^
60 °C	5.54 ± 0.62 ^a^	0.14 ± 0.01 ^b^	0.29 ± 0.03 ^b^	7.60 ± 0.75 ^a^	157 ± 3.94 ^a^	0.02 ± 0.03 ^a^

Note: Lyo, lyophilized reference control. Different superscript lowercase letters within a column indicate significant differences among hot-air drying temperatures according to Dunn’s post hoc test with Holm adjustment (*p* < 0.05); values sharing at least one letter do not differ significantly. The lyophilized reference was excluded from pairwise comparisons among hot-air drying temperatures.

## Data Availability

The data presented in this study are included in the article. Additional raw data supporting the findings of this study, including fatty acid methyl ester profiles and calculated fatty acid profile-derived indices, are available from the corresponding author upon reasonable request.

## Abbreviations

The following abbreviations are used in this manuscript:

FAME	fatty acid methyl ester
GC-FID	gas chromatography with flame ionization detection
SFA	saturated fatty acid
MUFA	monounsaturated fatty acid
PUFA	polyunsaturated fatty acid
IA	index of atherogenicity
IT	index of thrombogenicity
HH	hypocholesterolemic/hypercholesterolemic ratio
UI	unsaturation index
TFA	total trans fatty acid
PCA	principal component analysis
TBARS	thiobarbituric acid-reactive substances
